# Supplementary material for: Spatial genetic structure across a hybrid zone between European rabbit subspecies
Source: PeerJ. 2014 Sep 30;2:e582. doi: 10.7717/peerj.582 (PMC4183957; doi:10.7717/peerj.582)
Supplement: Table S1 — Rabbit localities sampled in this work, number of individuals analyzed, geographical coordinates and subspecies occurring in each locality according to it its natural distribution range. Numbers correspond to those indicated in Fig. 1. [file peerj-02-582-s001.pdf]

Table 1. Rabbit localities sampled in this work, number of individuals analysed, geographical coordinates and subspecies extant in each locality, according to its natural distribution range.

	Code	Locality	*n*	Longitude	Latitude	Subspecies
1.	Mallorca	Bunyola	14	2°55'3,72"	39°37'36,33"	*O. c. cuniculus*
2.	Lérida	L'Urgell y La Segarra	50	1°2'8,98"	41°45'3,69"	*O. c. cuniculus*
3.	Valencia	Vall d'Albadia, Finca La Amistad	18	0°30'39,63"	38°59'4,80"	*O. c. cuniculus*
4.	La Rioja	Logroño	19	2°9'20,04"	42°27'14,00"	*O. c. cuniculus*
5.	Galicia	Lugo	27	8°0'37,26"	42°46'48,27"	*O. c. cuniculus*
6.	Valladolid	Río Eresma y Río Cega	16	4°56'37,21"	41°27'9,93"	*O. c. cuniculus*
7.	Madrid1	Río Guadarrama, Finca Los Molinillos	51	3°54'39,98"	40°24'16,79"	hybrid zone
8.	Madrid2	Ajalvir	7	3°29'37,47"	40°34'19,98"	hybrid zone
9.	Madrid3	Aranjuez, Finca La Flamenca	2	3°36'12,58"	40°1'54,88"	hybrid zone
10.	Cuenca	Pozorrubio de Santiago	42	3°0'10,88"	39°50'23,00"	hybrid zone
11.	Toledo1	Toledo (Finca 1)	26	3°58'55,09"	39°52'55,03"	hybrid zone
12.	Toledo2	Toledo (Finca 2)	33	3°58'55,09"	39°52'55,03"	hybrid zone
13.	Toledo3	Gálvez, Finca El Borril	24	4°16'28,29"	39°42'12,23"	hybrid zone
14.	Toledo4	Río Guadarrama	2	4°3'5,55"	39°57'4,403"	hybrid zone
15.	Toledo5	Azucaica	19	3°58'47,18"	39°52'53,34"	hybrid zone
16.	Toledo6	Sonseca	11	3°58'9,37"	39°40'39,53"	hybrid zone
17.	Ciudad Real1	Santa Cruz de Mudela	51	3°27'11,95"	38°38'2,69"	*O. c. algirus*
18.	Ciudad Real2	Campo de Montiel	27	2°52'25,76"	38°42'22,7"	*O. c. algirus*
19.	Ciudad Real3	Almodóvar del Campo, Finca La Garganta	50	4°33'5,37"	38°32'40,88"	*O. c. algirus*
20.	Albacete	Sierra de Alcaraz	25	2°13'5,44"	38°58'14,99"	*O. c. algirus*
21.	Cáceres1	Valencia de Alcántara	10	7°14'38,67"	39°24'57,74"	*O. c. algirus*
22.	Cáceres2	Santa Marta de Magasca	28	6°5'59,53"	39°29'59,81"	*O. c. algirus*
23.	Badajoz1	Hornachos	20	6°4'10,81"	38°33'18,38"	*O. c. algirus*
24.	Badajoz2	Azuaga, Finca Magoilla	29	5°40'40,12"	38°15'34,87"	*O. c. algirus*
25.	Jaén1	Andújar	15	4°4'37,63"	38°5'38,06"	*O. c. algirus*
26.	Jaén2	Vilches	2	3°32'28,96"	38°13'18,23"	*O. c. algirus*
27.	Jaén3	Marmolejo	22	4°10'19,55"	38°2'42,47"	*O. c. algirus*
28.	Sevilla1	Las Cabezas de San Juan	43	5°56'27,11"	36°58'52,80"	*O. c. algirus*
29.	Sevilla2	Estepa	32	4°52'34,15"	37°17'38,74"	*O. c. algirus*
30.	Cádiz	Medina Sidonia, Finca Martelilla y Marqués de Domecq	56	5°55'27,68"	36°27'37,15"	*O. c. algirus*
