# Supplementary material for: Spatial genetic structure across a hybrid zone between European rabbit subspecies
Source: PeerJ. 2014 Sep 30;2:e582. doi: 10.7717/peerj.582 (PMC4183957; doi:10.7717/peerj.582)
Supplement: Table S2 — N, number of samples; NA, number of alleles; Ho, observed heterozygosity; He, expected heteroygosity; FIS, inbreeding coefficient. [file peerj-02-582-s002.pdf]

Supplementary Table 2. Genetic diversity statistics for all the rabbit localities analysed. N = number of samples, N_A= number of alleles, H_o= observed heterozygosity, H_e= expected heteroygosity, F_{IS}= inbreeding coefficient.

		**Sat3**	**Sat4**	**Sat5**	**Sat7**	**Sat8**	**Sat12**	**Sat13**	**Sat16**	**Sol33**	**Sol44**
Mallorca											
n= 14	N_A	7	7	8	5	6	4	5	6	6	6
	A_R	5.82	5.69	6.55	4.96	4.95	3.55	4.46	5.05	4.54	5.73
	H_o	0.85	0.79	0.93	0.71	0.46	0.71	0.38	0.43	0.57	0.30
	H_e	0.73	0.79	0.79	0.79	0.62	0.61	0.70	0.72	0.58	0.73
	F_{IS}	-0.1	0.0	-0.1	0.1	0.3	-0.1	0.5	0.4	0.0	0.6
Lérida											
n= 50	N_A	12	13	14	10	8	8	8	6	8	9
	A_R	6.59	7.40	8.10	6.38	5.50	5.49	5.58	4.98	5.75	6.89
	H_o	0.85	0.65	0.83	0.77	0.74	0.84	0.88	0.56	0.80	0.83
	H_e	0.80	0.85	0.85	0.82	0.74	0.77	0.74	0.77	0.78	0.84
	F_{IS}	-0.04	0.25	0.04	0.07	0.00	-0.07	-0.17	0.28	-0.02	0.04
Valencia											
n= 18	N_A	9	13	12	10	13	4	5	6	10	9
	A_R	7.70	10.62	8.43	7.65	9.19	3.46	4.23	5.38	6.99	7.16
	H_o	0.65	0.33	0.67	0.89	0.89	0.50	0.42	0.81	0.83	0.78
	H_e	0.85	0.90	0.85	0.86	0.89	0.52	0.64	0.76	0.82	0.84
	F_{IS}	0.27	**0.66**	0.24	-0.01	0.03	0.07	0.39	-0.03	0.01	0.10
La Rioja											
n= 19	N_A	9	7	8	6	8	6	3	7	8	7
	A_R	7.19	5.80	6.93	4.89	5.47	5.53	2.86	5.89	5.39	6.13
	H_o	0.72	0.78	0.95	0.58	0.89	0.89	0.47	0.59	0.72	0.78
	H_e	0.84	0.74	0.85	0.71	0.75	0.79	0.56	0.72	0.67	0.82
	F_{IS}	0.17	-0.02	-0.09	0.21	-0.16	-0.10	0.18	0.22	-0.06	0.08
Galicia											
n= 27	N_A	9	15	7	8	11	4	8	9	8	11
	A_R	6.37	7.32	6.06	6.67	7.57	3.50	6.50	7.03	6.26	8.24
	H_o	0.30	0.50	0.80	0.74	0.56	0.59	0.73	0.53	0.68	0.69
	H_e	0.79	0.71	0.82	0.83	0.83	0.66	0.84	0.83	0.82	0.87
	F_{IS}	**0.63**	**0.31**	0.05	0.13	**0.35**	0.12	0.15	**0.39**	**0.19**	0.23
Valladolid											
n= 16	N_A	10	8	12	8	8	4	6	7	7	8
	A_R	8.96	5.53	9.94	6.52	6.52	3.45	4.34	5.77	5.53	6.03
	H_o	0.60	0.56	0.73	0.81	0.62	0.69	0.44	0.88	0.71	0.63
	H_e	0.87	0.55	0.88	0.79	0.75	0.60	0.50	0.78	0.75	0.74
	F_{IS}	0.36	0.01	0.22	0.00	0.22	-0.11	0.16	-0.09	0.09	0.18

Supplementary Table 2. Continued.

		Sat3	Sat4	Sat5	Sat7	Sat8	Sat12	Sat13	Sat16	Sol33	Sol44
Madrid1											
n= 51	N_A	14	15	18	10	18	7	9	8	12	14
	A_R	8.51	8.16	9.43	6.91	9.41	5.02	6.05	6.30	8.16	9.01
	H_o	0.71	0.67	0.82	0.72	0.69	0.70	0.59	0.48	0.60	0.82
	H_e	0.89	0.87	0.90	0.84	0.90	0.75	0.80	0.84	0.89	0.90
	F_{IS}	**0.22**	**0.24**	0.09	**0.16**	**0.24**	0.07	**0.27**	**0.44**	**0.33**	0.10
Cuenca											
n= 42	N_A	11	23	26	10	13	7	7	8	10	10
	A_R	8.17	10.27	10.46	7.09	8.31	4.77	5.06	5.98	6.77	8.02
	H_o	0.65	0.93	0.88	0.74	0.68	0.76	0.76	0.56	0.65	0.81
	H_e	0.87	0.92	0.92	0.84	0.88	0.71	0.75	0.74	0.75	0.88
	F_{IS}	0.27	0.00	0.05	0.14	**0.25**	-0.06	0.01	**0.26**	0.15	**0.09**
Toledo1											
n= 26	N_A	12	8	8	6	13	5	10	7	9	10
	A_R	8.82	6.67	6.11	5.24	8.15	4.20	6.84	5.21	6.80	7.03
	H_o	0.71	0.70	0.83	0.58	0.90	0.68	0.91	0.44	0.64	0.68
	H_e	0.89	0.84	0.79	0.77	0.85	0.69	0.81	0.69	0.84	0.83
	F_{IS}	**0.23**	0.19	-0.02	**0.26**	-0.04	0.04	-0.10	0.38	0.26	0.21
Toledo5											
n= 19	N_A	11	9	9	8	9	6	7	6	11	9
	A_R	8.84	6.96	7.30	6.98	6.07	4.53	5.39	5.80	8.51	7.73
	H_o	0.50	0.89	0.73	0.67	0.47	0.71	0.82	0.37	0.75	0.74
	H_e	0.89	0.82	0.82	0.85	0.76	0.62	0.75	0.82	0.87	0.87
	F_{IS}	**0.46**	-0.05	0.14	**0.24**	0.41	-0.11	-0.07	**0.57**	0.17	0.18
Toledo2											
n=33	N_A	13	14	10	8	17	6	10	6	11	15
	A_R	8.96	7.81	6.45	6.01	9.42	3.73	6.45	5.61	7.42	8.81
	H_o	0.58	0.41	0.70	0.82	0.84	0.63	0.78	0.48	0.69	0.79
	H_e	0.90	0.84	0.81	0.82	0.89	0.67	0.81	0.82	0.84	0.89
	F_{IS}	**0.37**	**0.53**	0.16	0.02	0.07	0.08	0.05	**0.42**	**0.20**	0.13
Toledo3											
n=24	N_A	9	19	12	7	15	6	7	6	11	13
	A_R	6.81	10.02	7.16	5.87	9.36	4.79	5.13	5.23	7.83	8.85
	H_o	0.56	0.96	0.71	0.92	0.96	0.71	0.83	0.64	0.76	0.79
	H_e	0.83	0.90	0.82	0.82	0.89	0.72	0.76	0.79	0.85	0.89
	F_{IS}	**0.36**	-0.04	0.16	-0.10	**-0.05**	0.03	-0.08	0.22	0.13	0.13

Supplementary Table 2. Continued.

		Sat3	Sat4	Sat5	Sat7	Sat8	Sat12	Sat13	Sat16	Sol33	Sol44
Toledo6											
n=11	N_A	9	6	10	8	9	7	5	7	7	11
	A_R	7.81	5.54	8.54	7.30	7.82	6.31	4.87	6.70	6.76	9.87
	H_o	0.73	0.60	0.91	0.91	0.91	0.91	0.82	0.70	0.70	0.90
	H_e	0.83	0.73	0.86	0.83	0.84	0.79	0.76	0.82	0.84	0.89
	F_{IS}	0.17	0.22	-0.01	-0.04	-0.04	-0.11	-0.03	0.19	0.22	0.04
Ciudad Real1											
n=51	N_A	14	16	14	12	16	7	11	9	13	14
	A_R	8.63	7.84	8.00	7.63	8.97	3.80	6.96	6.38	6.45	7.76
	H_o	0.66	0.78	0.68	0.82	0.67	0.52	0.80	0.66	0.50	0.79
	H_e	0.90	0.86	0.86	0.87	0.88	0.69	0.84	0.84	0.79	0.86
	F_{IS}	**0.27**	0.11	0.22	0.06	0.26	0.25	0.06	**0.22**	**0.38**	0.09
Ciudad Real2											
n=27	N_A	10	12	14	9	13	6	6	7	8	12
	A_R	8.26	7.95	9.04	7.02	8.81	4.26	5.14	6.35	6.32	7.73
	H_o	0.52	0.85	0.65	0.81	0.67	0.58	0.70	0.22	0.45	0.85
	H_e	0.88	0.84	0.89	0.86	0.89	0.70	0.75	0.82	0.79	0.85
	F_{IS}	**0.43**	0.01	**0.28**	0.07	0.27	0.20	0.08	**0.74**	**0.44**	0.02
Ciudad Real3											
n=50	N_A	13	19	18	9	15	6	10	10	11	13
	A_R	8.33	10.15	9.76	6.13	8.66	3.95	6.40	6.55	5.21	8.43
	H_o	0.67	0.87	0.76	0.77	0.78	0.63	0.81	0.56	0.40	0.78
	H_e	0.88	0.92	0.91	0.82	0.88	0.67	0.82	0.79	0.58	0.89
	F_{IS}	**0.26**	0.06	**0.18**	0.07	0.13	0.07	0.02	**0.29**	**0.31**	0.13
Albacete											
n=25	N_A	11	13	12	8	12	5	7	7	10	12
	A_R	7.76	8.74	7.91	6.74	8.05	4.10	5.95	4.96	7.51	7.62
	H_o	0.57	0.86	0.84	0.96	0.65	0.60	0.92	0.52	0.71	0.95
	H_e	0.87	0.87	0.87	0.83	0.85	0.61	0.82	0.74	0.83	0.85
	F_{IS}	**0.37**	0.04	0.05	-0.13	0.26	0.04	-0.10	0.32	0.17	-0.10
Cáceres1											
n=10	N_A	5	7	8	8	8	5	6	5	5	9
	A_R	4.67	6.76	7.36	6.97	7.33	5.00	5.76	4.78	4.71	8.54
	H_o	0.44	0.67	0.90	0.70	0.90	0.63	0.90	0.89	0.30	0.67
	H_e	0.63	0.80	0.84	0.71	0.81	0.56	0.80	0.70	0.55	0.86
	F_{IS}	0.35	0.22	-0.03	0.06	-0.07	-0.05	-0.08	-0.22	**0.49**	0.28
Cáceres2											
n=28	N_A	10	7	8	8	16	6	10	6	8	14
	A_R	7.21	5.62	6.09	6.47	9.63	3.98	7.60	4.95	6.37	8.57
	H_o	0.58	0.79	0.68	0.71	0.50	0.46	0.85	0.50	0.52	0.89
	H_e	0.82	0.80	0.76	0.82	0.90	0.51	0.87	0.75	0.79	0.88
	F_{IS}	0.31	0.04	0.12	0.15	**0.46**	0.12	0.04	0.35	**0.36**	0.01

Supplementary Table 2. Continued.

		Sat3	Sat4	Sat5	Sat7	Sat8	Sat12	Sat13	Sat16	Sol33	Sol44
Badajoz1											
n=20	N_A	8	10	15	7	15	5	10	8	7	15
	A_R	6.88	5.45	8.75	5.34	9.02	4.52	7.73	6.10	5.56	9.34
	H_o	0.80	0.50	0.75	0.60	0.90	0.70	0.80	0.45	0.70	0.85
	H_e	0.83	0.69	0.83	0.65	0.87	0.71	0.86	0.79	0.77	0.89
	F_{IS}	0.06	0.29	0.13	0.11	-0.01	0.04	0.09	**0.45**	0.11	0.07
Badajoz2											
n=29	N_A	12	8	15	7	13	7	10	7	6	11
	A_R	8.35	5.93	9.27	5.67	8.24	4.53	7.79	6.06	5.26	8.28
	H_o	0.93	0.66	0.72	0.95	0.86	0.72	0.93	0.62	0.69	0.83
	H_e	0.88	0.76	0.88	0.78	0.87	0.68	0.87	0.80	0.78	0.88
	F_{IS}	-0.04	0.16	0.20	-0.20	0.02	-0.05	-0.05	0.24	0.13	0.08
Jaén1											
n=15	N_A	10	9	14	7	12	5	10	8	3	7
	A_R	7.89	7.14	10.32	5.84	8.99	4.06	7.28	7.05	3.00	6.12
	H_o	0.69	0.62	0.93	0.80	0.67	0.47	0.67	0.36	0.45	0.67
	H_e	0.80	0.78	0.91	0.80	0.88	0.63	0.80	0.82	0.57	0.77
	F_{IS}	0.17	**0.25**	0.01	0.04	0.27	0.30	0.21	**0.59**	0.24	0.18
Jaén3											
n=22	N_A	13	7	15	9	12	5	10	7	6	12
	A_R	8.65	5.18	9.44	7.70	8.37	3.80	8.20	5.82	5.24	8.49
	H_o	0.76	0.84	0.72	0.80	0.76	0.90	0.75	0.26	0.39	0.70
	H_e	0.88	0.75	0.88	0.86	0.88	0.67	0.87	0.76	0.75	0.86
	F_{IS}	0.16	-0.09	0.21	0.09	0.16	-0.32	0.17	**0.67**	**0.51**	0.21
Sevilla1											
n=43	N_A	13	10	17	13	14	5	10	8	10	14
	A_R	7.90	5.41	8.49	6.76	8.52	3.96	7.69	5.92	5.46	7.84
	H_o	0.83	0.67	0.86	0.76	0.79	0.58	0.81	0.53	0.60	0.79
	H_e	0.86	0.68	0.88	0.80	0.89	0.62	0.87	0.81	0.65	0.86
	F_{IS}	0.05	0.02	0.03	**0.05**	0.12	0.07	0.08	**0.35**	0.09	0.09
Sevilla2											
n=32	N_A	8	15	15	11	13	7	11	10	13	14
	A_R	6.64	9.07	9.91	7.00	8.20	5.90	8.08	6.64	6.88	8.45
	H_o	0.76	0.73	0.88	0.81	0.75	0.74	0.82	0.60	0.35	0.81
	H_e	0.83	0.87	0.91	0.82	0.84	0.79	0.86	0.80	0.69	0.87
	F_{IS}	0.11	0.19	0.06	0.04	0.14	0.08	0.08	0.27	**0.51**	0.08
Cádiz											
n=56	N_A	12	11	18	9	14	6	10	10	10	15
	A_R	6.54	6.95	9.08	6.08	8.90	4.26	7.46	6.20	4.80	7.42
	H_o	0.67	0.58	0.83	0.71	0.70	0.73	0.88	0.47	0.53	0.76
	H_e	0.79	0.82	0.90	0.78	0.90	0.66	0.87	0.79	0.58	0.84
	F_{IS}	0.16	**0.30**	0.09	0.11	**0.23**	-0.09	-0.01	**0.42**	0.09	0.10

F_{IS} values in bold represent significant deviations form Hardy-Weinberg equilibrium, after Bonferroni correction
