# Supplementary material for: Spatial genetic structure across a hybrid zone between European rabbit subspecies
Source: PeerJ. 2014 Sep 30;2:e582. doi: 10.7717/peerj.582 (PMC4183957; doi:10.7717/peerj.582)
Supplement: Table S3 — N, number of samples; NA, number of alleles; Ho, observed heterozygosity; He, expected heteroygosity; FIS, inbreeding coefficient. [file peerj-02-582-s003.pdf]

Supplementary Table 3. Genetic diversity statistics for the rabbit populations inferred in BAPS. N = number of samples, N_A = number of alleles, H_o = observed heterozygosity, H_e = expected heteroygosity, F_{IS} = inbreeding coefficient.

		Sat3	Sat4	Sat5	Sat7	Sat8	Sat12	Sat13	Sat16	Sol33	Sol44
K-BAL											
n= 14	N_A	7	7	8	5	6	4	5	6	6	6
	A_R	4.64	4.84	5.33	4.64	3.96	3.19	3.89	4.29	3.62	4.82
	H_o	0.85	0.79	0.93	0.71	0.46	0.71	0.38	0.43	0.57	0.30
	H_e	0.73	0.79	0.79	0.79	0.62	0.61	0.70	0.72	0.58	0.73
	F_{IS}	-0.12	0.04	-0.13	0.14	0.29	-0.14	0.48	0.44	0.05	0.62
K-NE											
n= 52	N_A	12	12	15	10	8	8	8	6	8	9
	A_R	5.29	5.98	6.29	5.18	4.59	4.57	4.50	4.45	4.77	5.69
	H_o	0.83	0.65	0.81	0.76	0.73	0.80	0.83	0.57	0.78	0.81
	H_e	0.80	0.86	0.86	0.82	0.74	0.76	0.74	0.77	0.78	0.85
	F_{IS}	-0.03	0.25	0.06	0.08	**0.02**	-0.05	-0.12	0.28	0.00	0.06
K-N											
n= 21	N_A	8	10	8	6	8	6	4	7	7	7
	A_R	5.61	5.17	5.60	4.04	4.67	4.83	2.85	4.88	3.84	5.06
	H_o	0.65	0.80	0.95	0.57	0.90	0.85	0.47	0.53	0.60	0.70
	H_e	0.83	0.76	0.84	0.71	0.77	0.79	0.52	0.76	0.63	0.80
	F_{IS}	0.24	-0.03	-0.11	0.21	-0.15	-0.05	0.12	**0.33**	0.08	0.15
K-CU											
n= 14	N_A	4	15	12	6	7	3	6	6	5	4
	A_R	3.82	7.79	6.43	5.21	6.15	2.98	3.76	5.13	4.36	4.00
	H_o	0.67	1.00	0.86	0.63	0.67	0.67	0.50	0.57	0.43	0.60
	H_e	0.72	0.91	0.83	0.77	0.76	0.64	0.49	0.78	0.70	0.70
	F_{IS}	0.11	-0.06	0.00	0.26	0.22	0.02	0.03	0.33	0.46	[illegible]
K-ZH											
n= 457	N_A	20	34	29	15	26	11	16	15	23	[illegible]
	A_R	6.91	6.85	6.94	6.09	7.37	3.86	5.50	5.50	6.35	[illegible]
	H_o	0.61	0.74	0.77	0.79	0.74	0.65	0.77	0.54	0.59	[illegible]
	H_e	0.91	0.89	0.90	0.88	0.92	0.72	0.83	0.85	0.88	[illegible]
	F_{IS}	**0.33**	**0.18**	**0.16**	**0.10**	**0.20**	**0.09**	**0.08**	**0.37**	**0.33**	[illegible]
K-S1											
n=206	N_A	17	18	20	14	20	8	15	17	13	[illegible]
	A_R	6.05	5.43	7.10	5.22	6.80	3.69	6.21	5.38	4.31	[illegible]
	H_o	0.76	0.62	0.82	0.76	0.74	0.66	0.84	0.53	0.56	[illegible]
	H_e	0.87	0.81	0.91	0.80	0.90	0.67	0.88	0.83	0.69	[illegible]
	F_{IS}	**0.12**	**0.23**	0.10	**0.05**	**0.18**	0.03	0.04	**0.37**	**0.19**	[illegible]

F_{IS} values in bold represent significant deviations form Hardy-Weinberg equilibrium, after Bonferroni correctio

Table S
sample
inbreec

K-BAL
n= 14

K-NE
n= 52
